# The complete chloroplast genome of *Callicarpa bodinieri* (Lamiales, Lamiaceae), an ornamental and medicinal plant from Chongqing, China

**DOI:** 10.1080/23802359.2021.1875932

**Published:** 2021-03-24

**Authors:** Chao-Ying Wang, Ji-Hong Luo, Hui-Liu He, Qian Wang

**Affiliations:** a Chongqing City Management College, Chongqing, China; b Chongqing Key Laboratory of Plant Resource Conservation and Germplasm Innovation, Institute of Resources Botany, School of Life Sciences, Southwest University, Chongqing, China

**Keywords:** *Callicarpa bodinieri*, chloroplast genome, Lamiaceae, phylogenetic analysis

## Abstract

Plants classified to the genus *Callicarpa* L. have important medicinal and ornamental value. Here, we report and characterize the complete chloroplast (cp) genome of *C. bodinieri* to provide molecular basis for the further studies on the phylogeny analysis of this genus. The cp genome is 154,183 bp in length and is organized with a typical quadripartite structure, containing two inverted repeats of 25,701 bp separated by a large single-copy region of 84,956 bp and a small single-copy region of 17,825 bp. The cp genome of *C. bodinieri* contains 112 distinct genes, including 78 protein-coding, 30 tRNAs and 4 rRNAs genes. The phylogenetic analysis showed that *C. bodinieri* is fully resolved in a clade with *C. nudiflora*, sister to the clade of *C. formosana* and *C. longifolia* var. *floccosa*.

*Callicarpa* L. contains about 140 species worldwide, and is mainly distributed in tropical and subtropical Asia (Chen and Michael 1994). The genus was traditionally assigned to the Verbenaceae (Chen and Michael 1994), however recent molecular studies placed it in the Lamiaceae (Li et al. 2016). *Callicarpa bodinieri* H. Lév. 1911 is a traditional Chinese medicine herb with anti-inflammatory activity (Gao et al. 2020). It displays luminous purple berries and has a long fruiting period, which makes it an ideal ornamental plant. Chloroplast genomes are important sources for phylogenetic analyses and plant molecular identification (Sun et al. 2020). In this study, we report the chloroplast (cp) genome of *C. bodinieri* and its phylogenetic relationship within the Lamiaceae.

Fresh leaves of *C. bodinieri* was collected from Pengshui County of Chongqing, China (N28°59′18.71″, E107°52′55.25″, 847 m). The voucher specimen (CY Wang PS 20191001) was deposited in the herbarium of Southwest University (SWCTU, Qian Wang, wangqian123@swu.edu.cn). The total genomic DNA was extracted and used for sequencing on Illumina HiSeq 4000 platform at the Beijing Novogene Bioinformatics Technology Co., Ltd. (Nanjing, China). To obtain the complete cp genome, *de novo* assembly of the raw data was performed using SPAdes (Bankevich et al. 2012). The complete genome sequence was annotated using PGA (Qu et al. 2019) with manual adjustments. The sequence cp genome was deposited in GenBank (accession number MW149077).

The cp genome of *C. bodinieri* is 154,183 bp in length and has a typical quadripartite angiosperm structure, containing two inverted repeats (IRs) of 25,701 bp, a large single-copy (LSC) region of 84,956 bp, and a small single-copy (SSC) region of 17,825 bp. The GC content of the whole cp genome is 35.9%. The cp genome of *C. bodinieri* contains 112 distinct genes, including 78 protein-coding (PCGs), 30 transfer RNA (tRNA), and 4 ribosomal RNA (rRNA) genes. Of these genes, 16 are duplicated, including 5 PCGs, 7 tRNA and 4 rRNA genes. In addition,15 of these genes have one intron, and 3 have two introns (*rps12*, *ycf3*, and *clpP*).

To determine the phylogenetic position of *C. bodinieri* with related species, the whole cp genome sequences from 20 species of Lamiaceae, Paulowniaceae, Phymaceae and Oleaceae (outgroup) were downloaded from GenBank. The sequences were aligned with MAFFT version 7.0 (Katoh and Standley 2013). The maximum-likelihood (ML) phylogenetic trees were reconstructed using RAxML (Stamatakis 2014) with the GTR Gamma and 1,000 bootstrap replicates. The result showed that *C. bodinieri* was resolved in a fully supported clade with three other species of *Callicarpa* (Figure 1). The result support the placement of *Callicarpa* in Lamiaceae, which is consistent with the research of Li et al. (2016).

**Figure 1.**
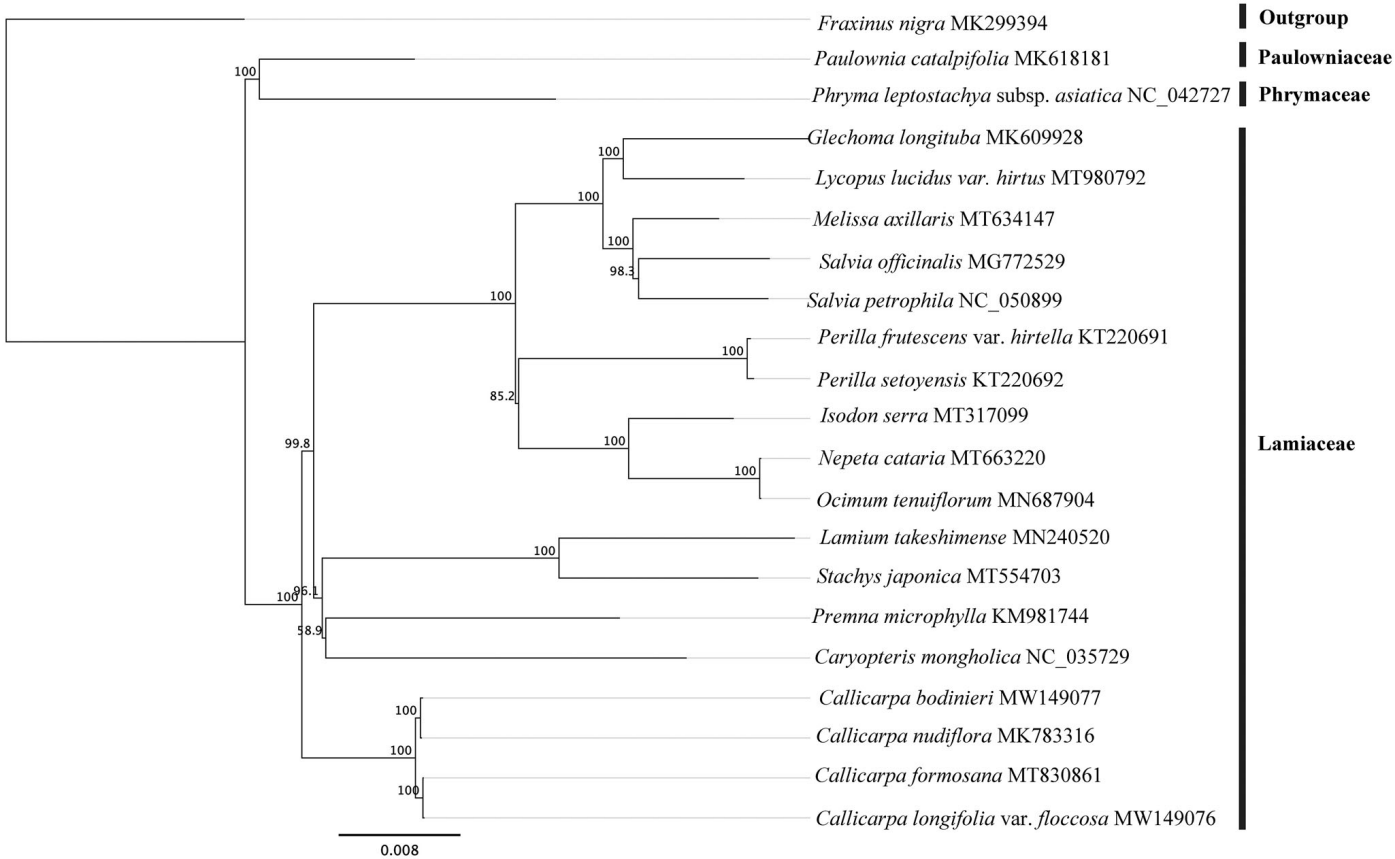
Phylogenetic tree based on 21 complete chloroplast genome sequences. Numbers at nodes correspond to ML bootstrap percentages (1000 replicates). All the sequences are available in GenBank, with the accession numbers listed to the right of their scientific names.

## Data Availability

Chloroplast data supporting this study are openly available in GenBank at nucleotide database, https://www.ncbi.nlm.nih.gov/nuccore/MW149077, Associated BioProject, https://www.ncbi.nlm.nih.gov/bioproject/PRJNA687337, BioSample accession number at https://www.ncbi.nlm.nih.gov/biosample/SAMN17141367 and Sequence Read Archive at https://www.ncbi.nlm.nih.gov/sra/SRR13292319.
